# Identification of diagnostic markers and immune cell infiltration characteristics in antineutrophil cytoplasmic antibody-associated vasculitis by weighted gene co-expression network analysis

**DOI:** 10.1186/s40001-022-00666-3

**Published:** 2022-03-05

**Authors:** Mengdi Xia, Fen Zhao, Yongji Zhang, Zhihuang Zheng, Yun Zhou, Tong Liu

**Affiliations:** 1 Nanchong Key Laboratory of Basic Science & Clinical Research On Chronic Kidney Disease, Department of Nephrology, The Second Clinical Medical Institution of North Sichuan Medical College (Nanchong Central Hospital), Nanchong, China; 2 Shanxi Kidney Disease Institute, Department of Nephrology, Shanxi Provincial People’s Hospital, The Affiliated People’s Hospital of Shanxi Medical University, Taiyuan, China; 3 Department of Nephrology, Shanghai General Hospital, Shanghai Jiaotong University School of Medicine, Shanghai, China

**Keywords:** Antineutrophil cytoplasmic antibody-associated vasculitis (AAV), ANCA-associated glomerulonephritis, Hub gene, CIBERSORT, Immune infiltration

## Abstract

**Background:**

Antineutrophil cytoplasmic antibody-associated vasculitis (AAV) is a group of life-threatening systemic autoimmune diseases. The aim of this study was to determine the relationship between the AAV hub gene and immune cell infiltration, and its value for clinical disease treatment.

**Methods:**

We downloaded the microarray information of 37 AAV patients and 27 controls from Gene Expression Omnibus (GEO). Genes were classified into totally different modules exploitation weighted gene co-expression network analysis (WGCNA). AAV diagnostic indicators were screened and then assessed immune cell infiltration by the least absolute shrinkage and selection operator (LASSO) and CIBERSORT. Finally, Connectivity Map analysis was applied to predict possible AAV glomerulus injury improvement therapies.

**Results:**

WGCNA was developed and differentially expressed genes were classified into 6 modules, the black module was most tightly correlated to AAV. Among them, TIMP1 and FCER1G were most closely related to clinical features. Resting mast cells and monocytes emerged as having the foremost distinguished variations in AAV. C3AR1 and FCER1G were involved in AAV development by immune regulation. Connectivity Map analysis indicated the most significant compound was fisetin.

**Conclusions:**

The present study is that the initial to spot immune cell infiltration with microarray data of glomeruli in AAV, which provides novel proof and clues for additional analysis of the molecular mechanisms.

## Introduction

Antineutrophil cytoplasmic antibody (ANCA)-associated vasculitis (AAV) is a group of life-threatening systemic autoimmune diseases characterized by inflammation and destruction of small- and medium-sized blood vessels, including three types: microscopic polyangiitis (MPA), granulomatosis with polyangiitis (GPA), and eosinophilic granulomatosis with polyangiitis (EGPA) [1]. Similar to other autoimmune diseases, the mechanism by that ANCAs cause vasculitis involves ANCA-mediated excessive activation of immune cells consisting of innate and adaptive immune populations [2]. Activation or inactivation of various types of immune cells in AAV contributes to disease initiation and augmentation by regulating the suppression, maintenance, or promotion of immune responses [2, 3]. AAV is usually responsible for grievous kidney failure or pulmonary hemorrhage [1], especially for the kidney, with > 75% of AAV patients have renal involvement, which is called ANCA-associated glomerulonephritis (GN) [4, 5]. In that case, GPA and MPA are the major types in AAV, both leading a pauci-immune necrotizing glomerulonephritis [1]. As an important complication of AAV, ANCA-associated GN directly affects the clinical outcome of the patients [6]. Preventing or ameliorating kidney injury has become an essential part of AAV treatment [7]. Given the functionally distinct cell sorts that comprise the immune reaction, assessing immune infiltration and deciding whether or not variations within the composition of the immune infiltration will improve the event of novel therapy medicine to focus on these cells is vital in AAV and ANCA-associated GN [8]. Although advances in treatment methods and the prognosis of patients have improved over the past decades, there are still a relatively large number of patients who end up in End-Stage Renal Disease (ESRD) present with low eGFR and do not recover renal function [9] and a serious burden of morbidity and mortality in AAV [10]. Hence, there is a requirement for new strategies for exploring AAV.

Gene sequence technologies and bioinformatic analyses have been used in recent years for the identification of disease-related genes which could be used for prognostic biomarks and produced in future as therapeutic targets [11]. Therefore, tracking the biological changes in AAV at the genomic level is a valuable technique [12]. Although several previous studies had focused on genes closely associated with AVV [13, 14], transcriptomics at the cellular level was few reported. To our knowledge, few studies utilized bioinformatic analysis and immune cell infiltration to characterize kidney tissue in the context of AVV. The aim of this study was to contribute to further mechanistic studies of AAV by analyzing the relationship between the hub gene and immune infiltrating cells and their associated immunological processes in AAV kidney tissue.

## Materials and methods

### Data collection and preprocessing

The AAV RNA expression data were acquired from the Gene Expression Omnibus (GEO; http://www.ncbi.nlm.nih.gov/geo/). For differential expression analysis, data from GSE108109 and GSE104948 were employed. The GSE 108109 (GPL19983 platform, Affymetrix Human Gene 2.1 ST Array, http://dx.doi.org/10.1136/annrheumdis-2017-212935) contains 21 specimens, including 6 normal controls and 15 specimens of AAV. The GSE 104948 (GPL22945 platform, Affymetrix human genome U133 Plus 2.0 array, http://dx.doi.org/10.1136/annrheumdis-2017-212935) contains 43 specimens, including 21 normal controls and 22 AAV specimens. AAV samples were obtained from kidney tissue specimens from patients with confirmed ANCA-GN diagnosis after kidney puncture pathology, and healthy kidney tissue was obtained from living transplant donors. Among them, the types of AAV are GPA and MPA. A positive ANCA antibody was present in both AAV patients. In order to obtain the matrix of gene expression, R v3.6.1 was used to extract and sort data. The batch effect caused by study heterogeneity was eliminated using the “*ComBat module”* of the “*SVA package,”* which utilizes empirical “*Bayes”* methods. Background adjustments and data normalization were performed with the “*limma package”* [15].

### Identification of differentially expressed genes

We filtered the differentially expressed genes (DEGs) among AAVs and controls using the “*limma*” R package in the expressing results. The significance analysis of microarrays (SAM) was accustomed choose considerably altered genes with false discovery rate (FDR) < 0.05 and log2 fold change (FC) ≥ 0.5.

### Establishment of co-expression network

Based on the R package “*WGCNA*” the DEG co-expression network was designed [15]. The soft threshold power we selected was eight when 0.8 was the threshold of the correlation and a minimum of 10 genes in the modules were chosen. We have specified 0.2 as the cutting height threshold to combine possibly related modules.

### Detection of hub genes

Hub genes with gene significance higher than 0.6 and high module group members (MM) (weighted correlation index > 0.9) were considered in the module trait correlation analysis, demonstrating a significant correlation to certain clinical characteristics.

### GSEA-based enriched GO and pathway analysis

Gene Ontology (GO) analysis included biological process (BP), cellular component (CC), and molecular function (MF). Reactome is a primary database for the research of pathways. We selected BP to perform GO analysis, as well as Reactome to perform pathway analysis for the crucial module; it was accomplished in the "cluster Profiler" package via gene set enrichment analysis (GSEA). The cut-off parameters were the adjusted *P*-value < 0.05.

### Assessment of immune cell infiltration

To delete the null values, gene expression datasets had been analyzed. In the "impute" page [16], the missing value was completed with KNN; the format was established in compliance with the recognized CIBERSORT format, followed by the uploading of data to the CIBERSORT website (http://cibersort.stanford.edu/). We used the original CIBERSORT LM22 gene signature file to study datasets, which described 22 immune cell subtypes. Samples that fulfill the CIBERSORT *P* < 0.05 conditions were included with immune cells profiles.

### Analysis of differences among types of immune cell infiltration

We analyzed the main differential expression of different cell immune cell types using the differential analysis between the group AAV and control group. A linear model was used in the “*limma*” bundle and Bayesian method [17]. The cut-off standard was *P*-value < 0.05. Spearman's correlating coefficient was used to find a similarity among certain differentially expressed types of immune cells, in order to further see the association between these different immune cells types.

### LASSO model and receiver operating characteristic (ROC) curve analysis

A good predictive value and low correlation were extended to the least absolute shrinkage and selection operator (LASSO), which chose the best features for the high-dimensional data [18]. We also extracted the hub gene expression profile for building LASSO model by “*glmnet*” package (https://CRAN.R-project.org/package=glmnet) to separate AAV from control. Datasets were at random appointed to the training set (70%) and test set (30%). The “*pROC*” packet [15] was utilized for analyzing ROC curve in training and test set to assess the ability of the LASSO model to classify AAV.

### Statistical and CMap analysis

In the case of AAV, the online portal (http://v5.nephroseq.org) was used for the Spearman correlation analysis among hub genes and glomerular filtration rate (GFR) [19, 20] and serum creatinine (SCr) [21, 22]. Connectivity Map (CMap) (https://portals.broadinstitute.org/cmap) is an open source, linking disease, genes, and medicines through similar or opposite gene expression profiles [23]. A CMap analysis was applied to forecast possible AAV glomerulus injury improvement therapies.

## Results

### Bioinformatic analysis workflows and DEGs related to AAV

**Fig. 1:**
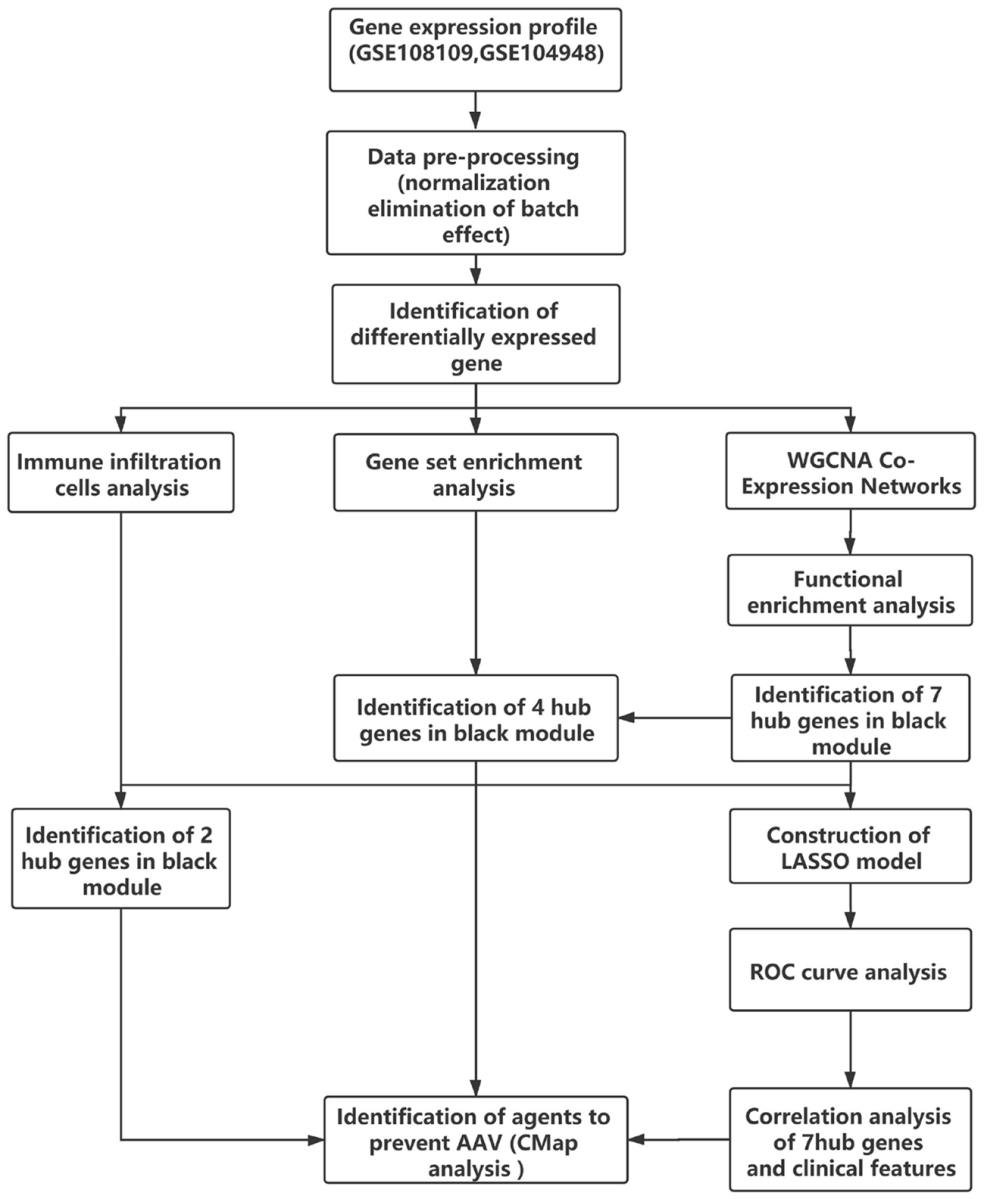
Workflows of our bioinformatic analysis

**Fig. 2:**
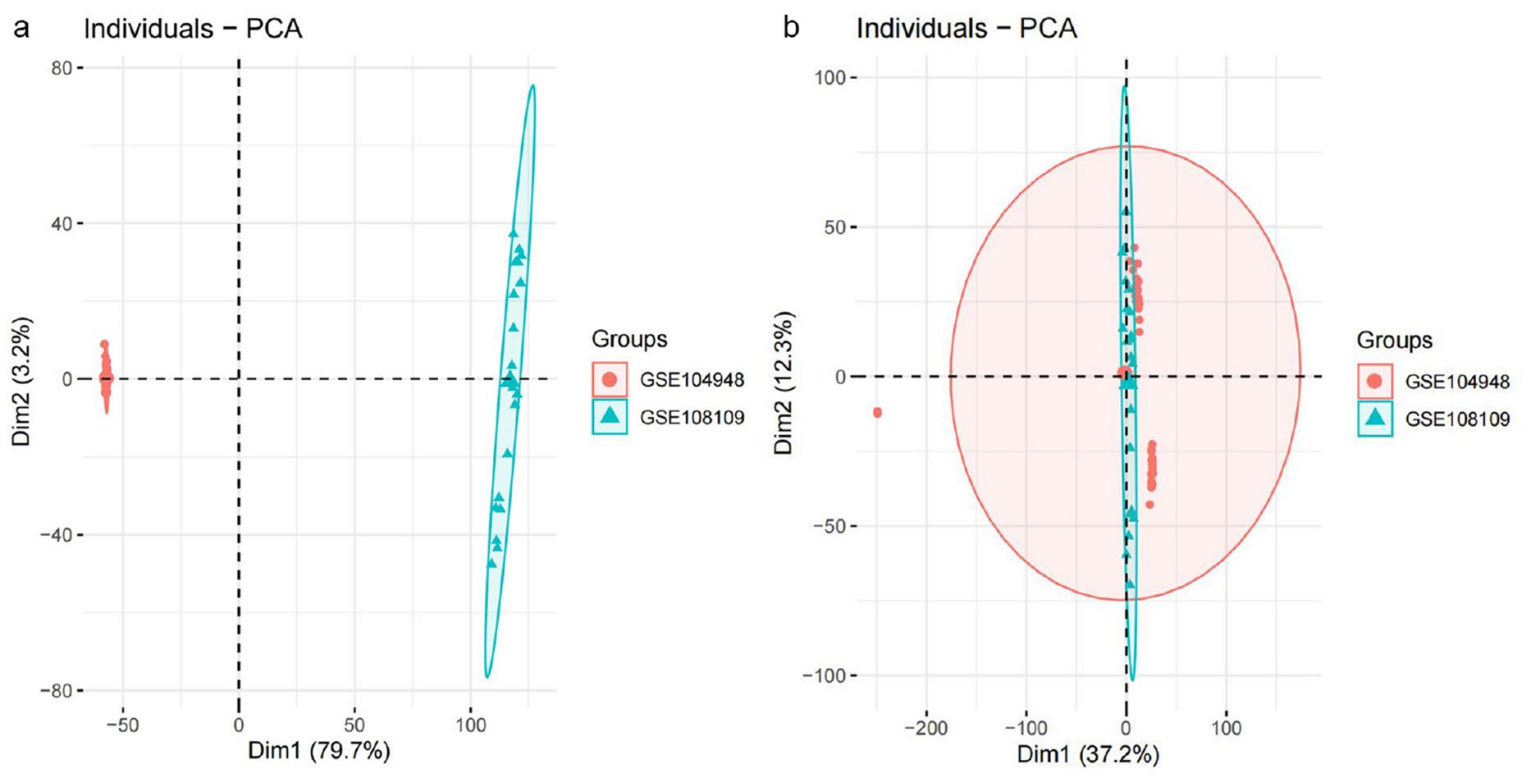
Principal component analysis (PCA) of the gene expression datasets. The scatter points show the samples on the base of the top two principal components of gene expression profiles (PC1 and PC2) without **a** and **b** removing the batch effect. The colors reflect samples in two different datasets

**Fig. 3:**
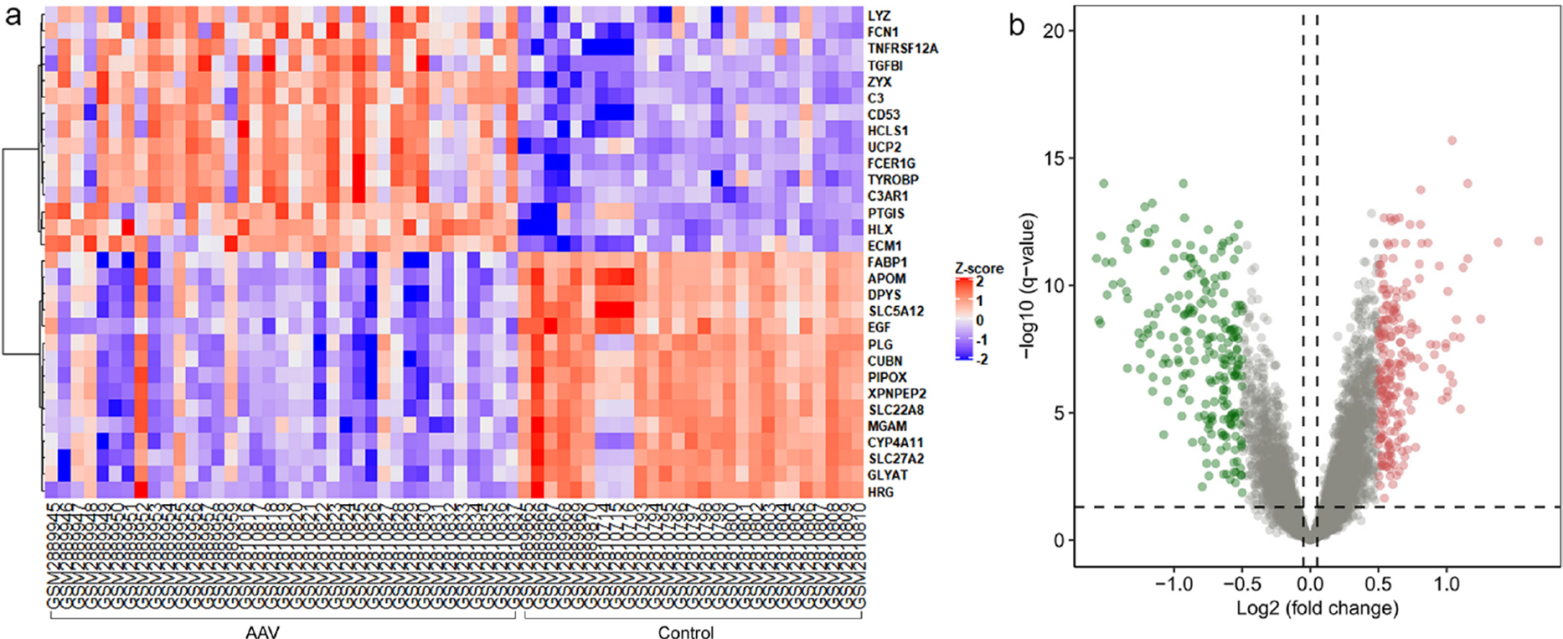
Difference analysis.** a** Heatmap (top 30 genes) of 445 DEGs screened by “*limma package.*” Red represent highly expressed genes and blue represent lowly expressed genes in AAV compared with normal controls. **b** Volcano plot analysis identifies DEGs. Red dots represent upregulated genes and green dots represent downregulated genes in AAV compared with normal controls. *DEG* differentially expressed gene; *AAV* ANCA-associated vasculitis

The workflows are shown in Fig. 1. After eliminating the batch effect by cross-platform from the microarray data (Fig. 2), 445 DEGs included in AAV were screened in the heatmap (Fig. 3a) by the limma package (adjusted p < 0.05, |logFC|≥ 0.5). 225 genes were downregulated and 220 genes were upregulated (Fig. 3b).

### Weighted gene co-expression network analysis (WGCNA) co-expression networks

**Fig. 4:**
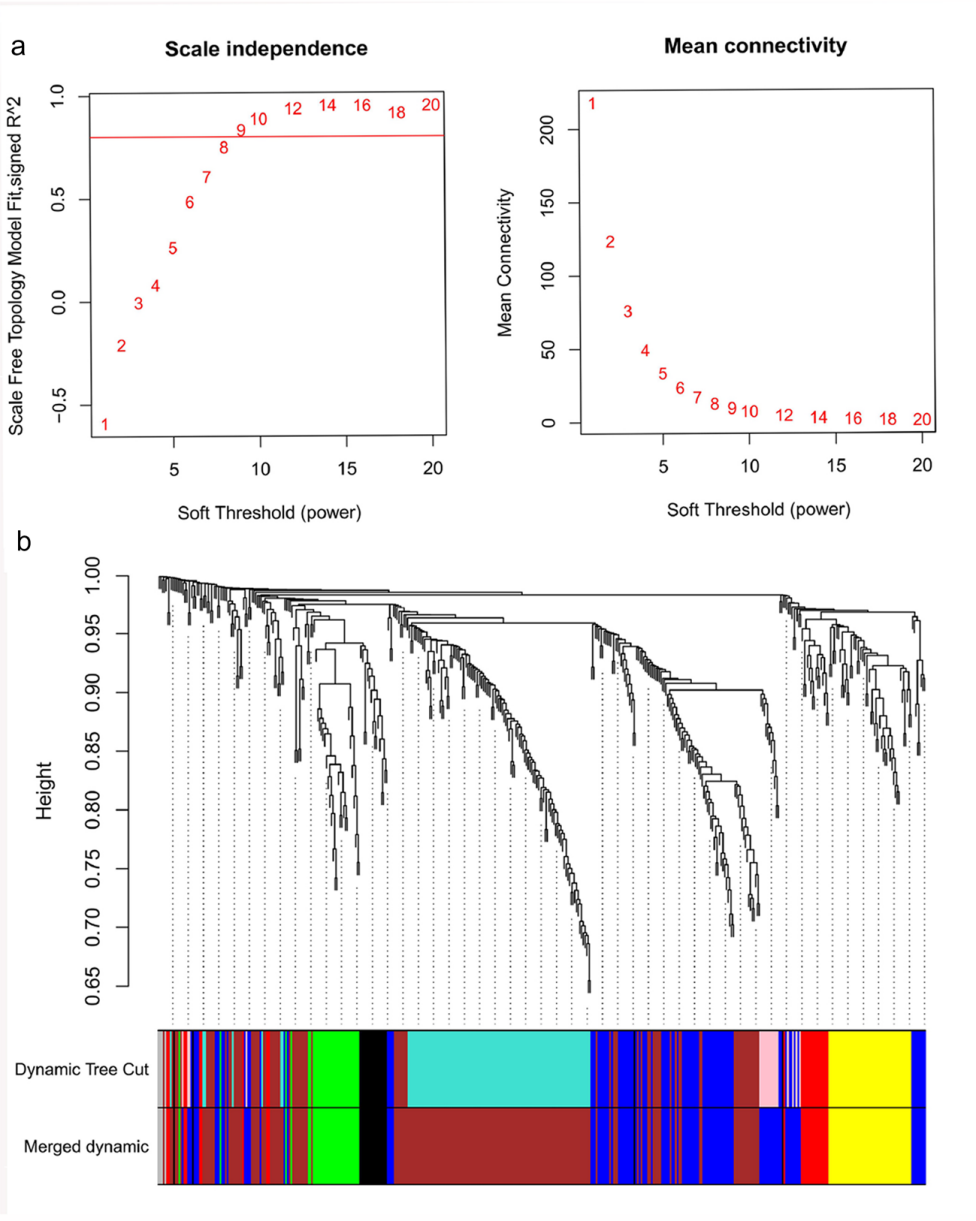
WGCNA revealed gene co-expression networks in the glomerulus of AAV.** a** Analysis of the scale-free fit index for various soft thresholding powers (Left) and analysis of the mean connectivity for various soft thresholding powers (Right); **b** Clustering dendrogram of DEGs related to the glomerulus of 37 AAV

The soft thresholding power was chosen as 10, while 0.8 was used as the correlation coefficient threshold (Fig. 4a). 6 co-expression modules were built by WGCNA analysis (Fig. 4b). The module comprised the majority of genes was brown, followed by black, green, and blue (Fig. 4b).

### AAV module trait correlations and Hub Genes identification

**Fig. 5:**
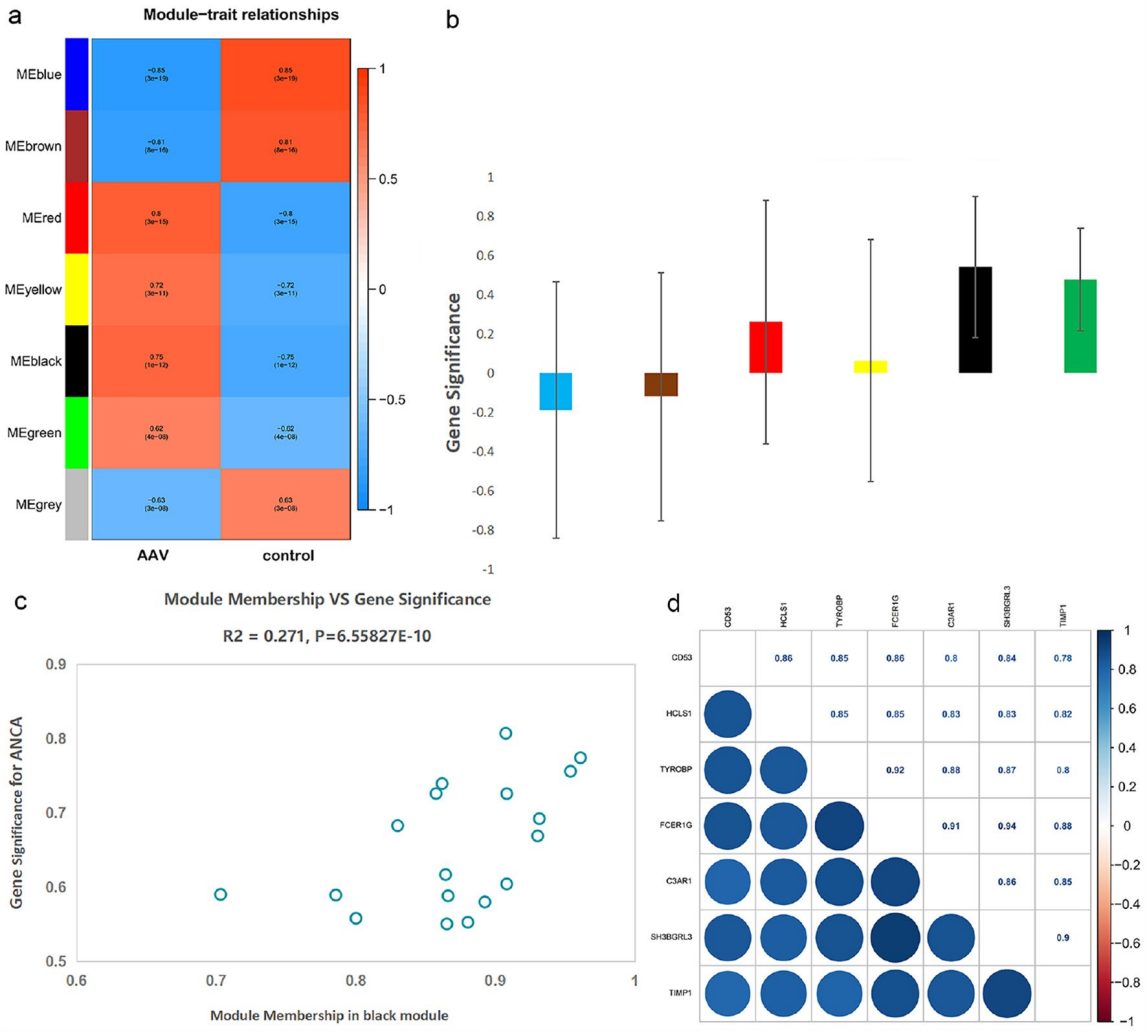
Main findings in the module trait correlations analyses.** a** Heatmap representing the relationship between modules and AAV (the correlation coefficient and corresponding P-value were present in each cell); **b** The module significance values of certain co-expression modules related to AAV (the module significance value expressed a description of the gene significance of all genes in each module, and different column colors represent different modules); **c** AAV gene significance in the black module (In the black module, one dot represents one gene.); **d** 7 hub genes in the black module were strongly associated with each other

The study of module trait correlations found that multiple modules were correlated to AAV (Fig. 5a). The summary of the significance of all genes associated with AAV in each module is shown in Fig. 5b. AAV (correlation coefficient = 0.75, P = 1E-12; Fig. 5a) was positively correlated to the black module, while the brown module was negatively correlated with AAV (correlation coefficient = -0.81, P = 8E-16; Fig. 5a). In the black module, Fig. 5c shows the significance of these genes. In black modules 7 genes (*TIMP1, FCER1G, SH3BGRL3, HCLS1, C3AR1, TYROBP,* and *CD53*) were identified as hub genes, according to GS > 0.6 and MM > 0.9 (Fig. 5c, d).

### Enrichment analysis of hub genes

**Fig. 6:**
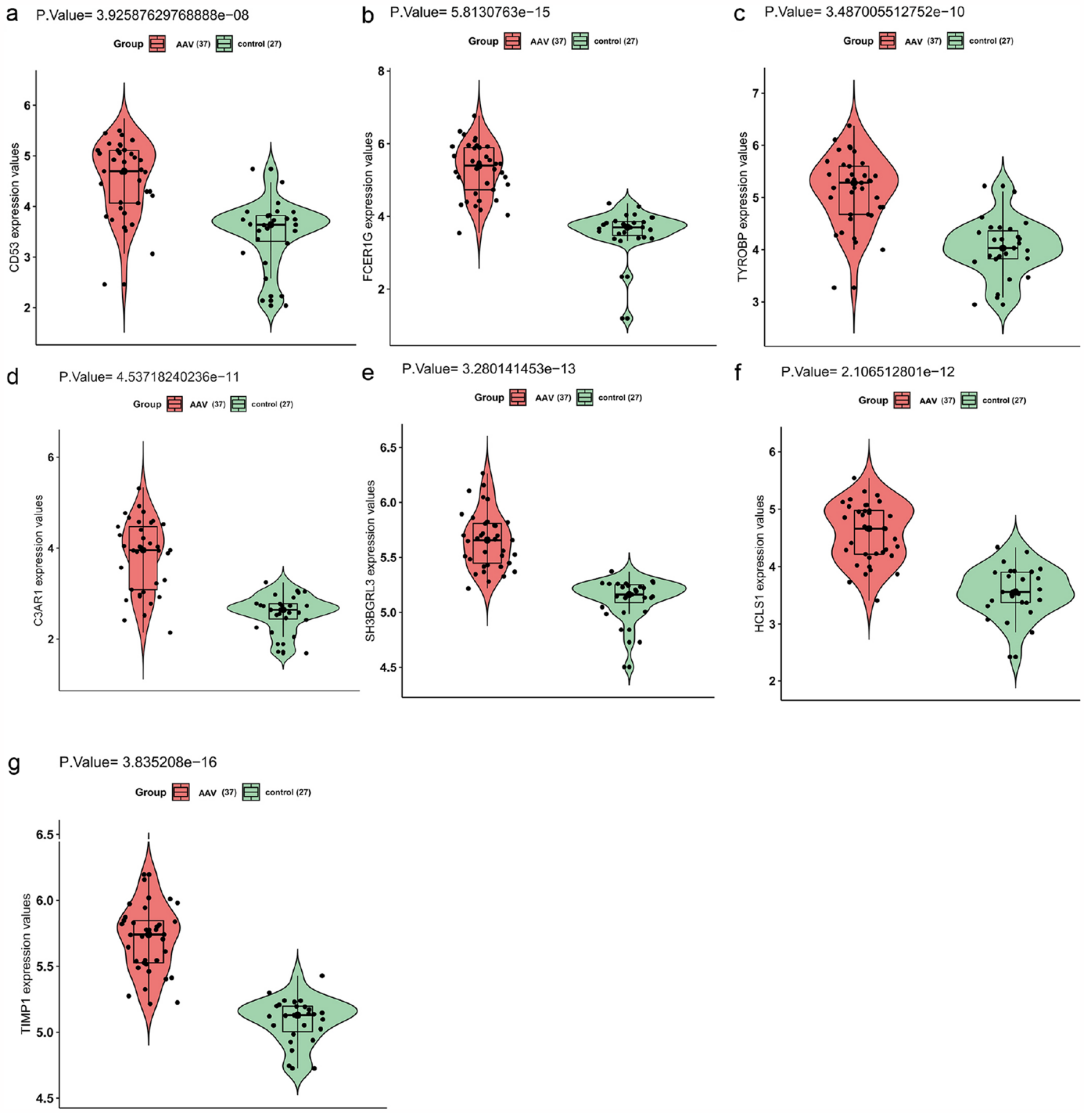
Expression of hub genes. (a–g) Expression levels of *CD53* **a**, *FCER1G* **b**, *TYROBP ***c**, *C3AR1* **d**, *SH3BGRL3* **e**, *HCLS1* **f**, and *TIMP1* **g** were significantly increased in AAV patients

**Fig. 7:**
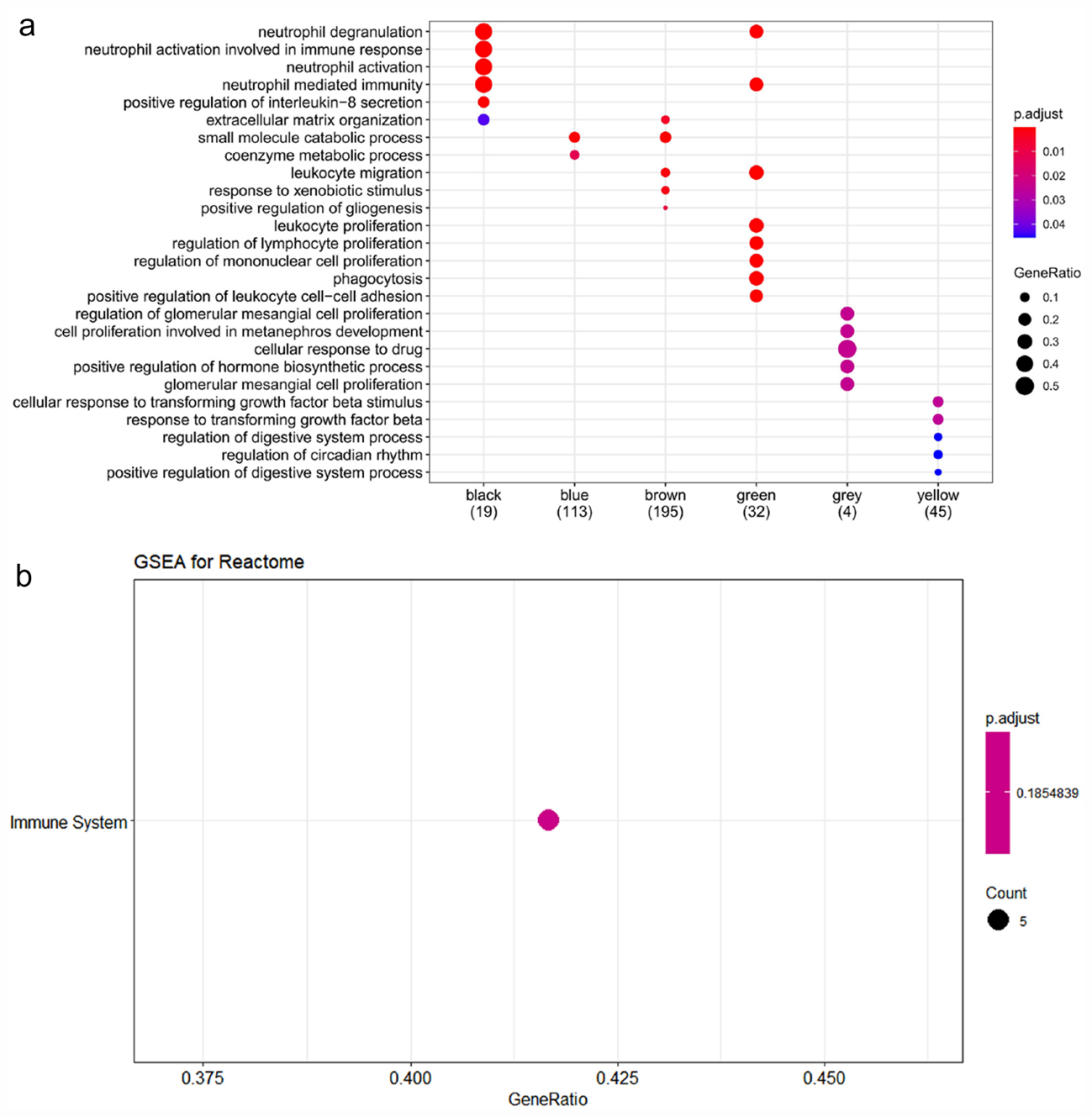
Enrichment analysis of hub genes. **a** The biological processes in module genes increasingly raise the significance of enrichment from blue to red, and point size shows the number of differential genes in the functional pathway. **b** The pathway of black module enrichment

In the black module of the merged dataset, the expression of 7 hub genes, including *CD53* (Fig. 6a), *FCER1G* (Fig. 6b), *TYROBP* (Fig. 6c), *C3AR1* (Fig. 6d), *SH3BGRL3* (Fig. 6e), *HCLS1* (Fig. 6f), and *TIMP1* (Fig. 6g), was significantly increased in the AAV patients. Module function enrichment analysis demonstrated the substantial involvement of the black module genes (Fig. 7a) in biological processes associated with immunity mediated by neutrophils. Pathway analysis revealed that the only pathway enriched in the black module was the immune pathway (Fig. 7b).

### GSEA-based analysis of all detected genes

**Fig. 8:**
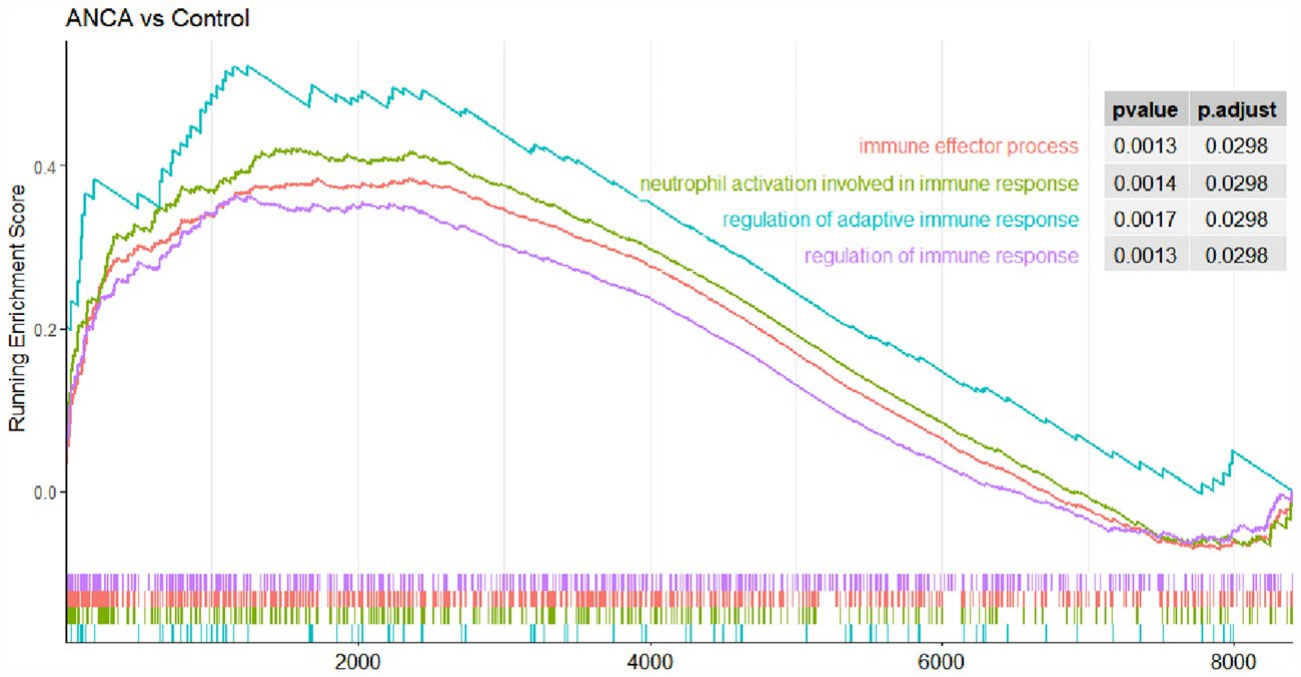
GSEA-based analysis of the main enrichment processes in AAV

GSEA was conducted to detect genes with a statistically significant difference in AAV. Cell activation in the immune process included the most important enriched genes positively associated with the AAV (Fig. 8). Further evidence showed that the immune process was the most significant cause of AAV. Table1 shows the most critical pathways after the black module GO screening. Among the 7 hub genes screened by the black module, the genes involved in the immune effector process were *FCER1G, C3AR1, TYROBP, and CD53*.

**Table 1:** GO analysis in the black module filtered with meaningful 4 items of BP

ID	Description	NES	P-value	Core Enrichment
GO:0,050,896	response to stimulus	1.73	0.02	*FCER1G/C3AR1/LYZ/TYROBP/CD53/TGFBI/HCLS1/FCN1/RNASE6/APBB1IP/TGM2/IFI30*
GO:0,043,170	macromolecule metabolic process	1.79	0.01	*FCER1G/C3AR1/LYZ/TYROBP/TGFBI/HCLS1/FCN1/RNASE6*
GO:0,002,252	immune effector process	1.71	0.03	*FCER1G/C3AR1/LYZ/TYROBP/CD53/FCN1/RNASE6/APBB1IP*
GO:0,071,704	organic substance metabolic process	1.64	0.04	*FCER1G/C3AR1/LYZ/TYROBP/TGFBI/HCLS1/FCN1/RNASE6*

*NES* normalized enrichment score

### Screening and verification of the potential diagnostic marker

**Fig. 9:**
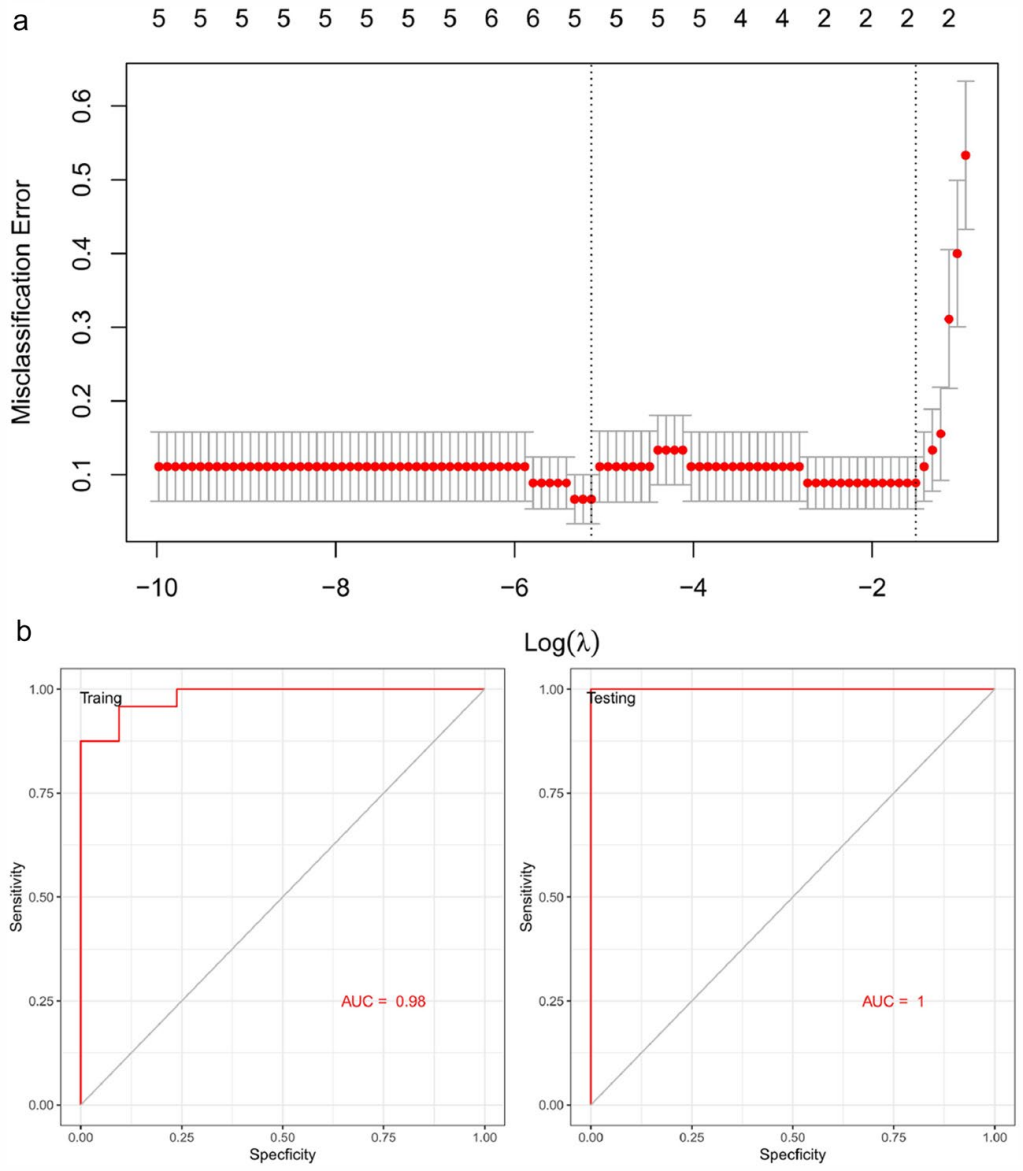
A model for predicting and verification of AAV. **a** LASSO model. **b** ROC curves analysis of train and test set

We extracted the expression profile for the LASSO model of the hub genes (Fig. 9a). 2 hub genes (*TIMP1* and *FCER1G*) with non-zero regression coefficients had been defined using the LASSO procedure, and the value of lambda. 1se = 0.103313. The index model of the genes was as follows: index = *TIMP1* *(0.4335136) + FCER1G* (0.1505385). The ROC (Fig. 9b) study revealed a 2-gene model AUC of 0.98 for the training and 1.0 for the test sample.

### Performance of CIBERSORT

**Fig. 10:**
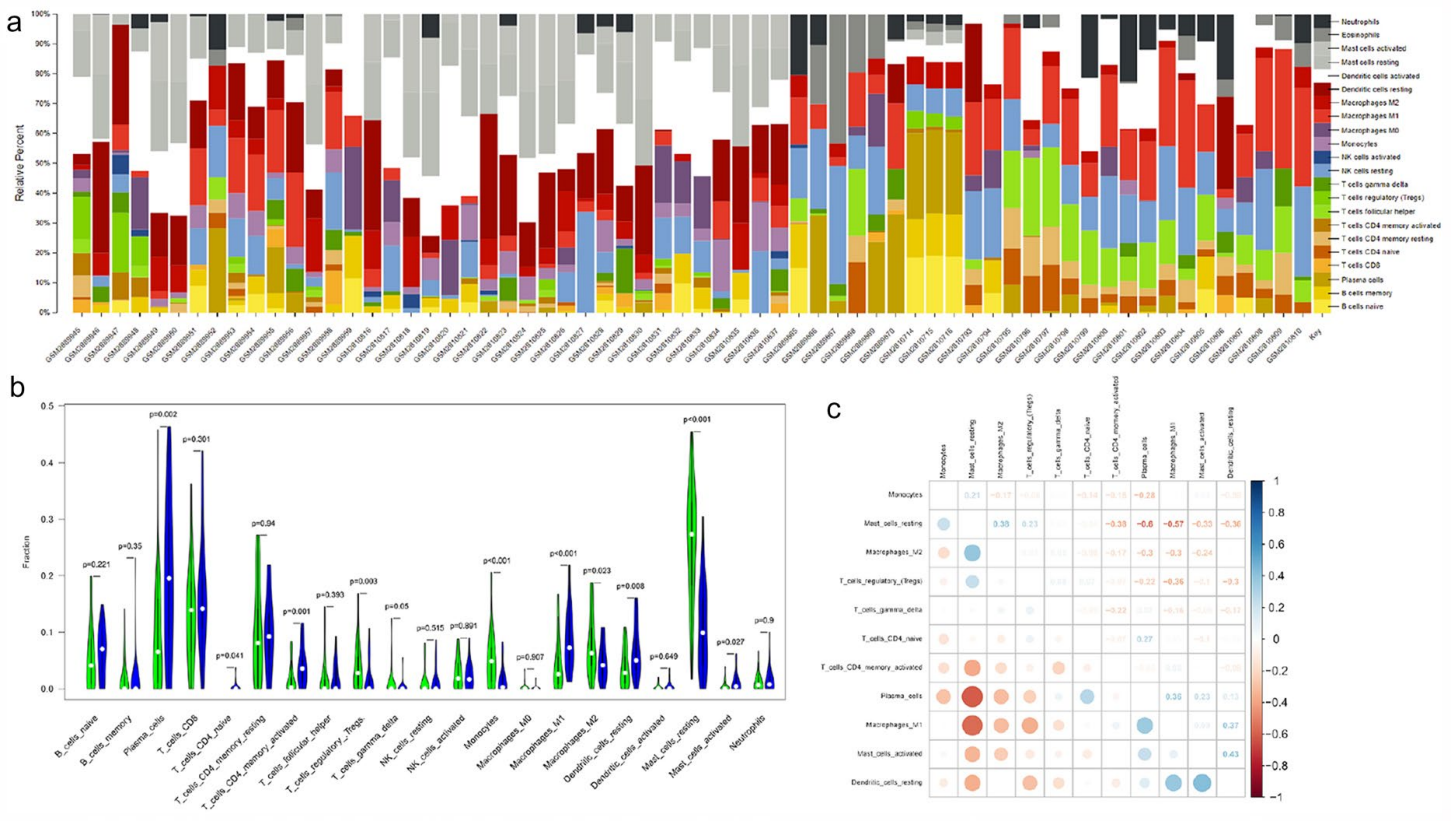
Landscape of immune infiltration in AAV.** a** Bar charts of 22 immune cell ratios in AAV and normal tissues. **b** Differential expression of different types of immune cells between AAV (green) and normal tissues (blue). **c** Correlation matrix of 11 types of immune cell proportions. Data were collated by using R package “*tidyverse*” (version 1.2.1). R package “*ggpubr*” (version 0.1.8) was used for T test. Results visualization was performed by using R package “*ggplot2*” (version 3.1.0). Correlation analysis and visualization were showed by using R package “*corrplot*” (version 0.84)

Figure 10a shows the ratio of immune cells in 37 renal tissues, among which the most infiltrated cells in AAV were mast cells. It showed the difference in the expression of the immune infiltration cells in the AAV and control groups in Fig. 10b. 11 immunocellular types were differential expression, namely plasma cell T, naive CD4 T cell, activated memory CD4 T cell, regulatory T cell (Tregs), γδT cell, monocyte, M1 macrophage, M2 macrophagic, resting dendritic cell, resting mast cell, and activated mast cell. Tregs, γδT cells, monocytes, M2 macrophages, and resting mast cells were upregulated in ANCA-associated GN tissue. Among them, the increase in resting mast cells and monocytes was the most significant. Plasma cells, naive CD4 T cells, activated memory CD4 T cells, M1 macrophages, resting dendritic cells, and activated mast cells were downregulated. The relation between these differentially expressing immune cell types is shown in Fig. 10c. Resting mast cells were negatively related to with plasma cells and M1 macrophages (r =  − 0.6 and r =  − 0.57, respectively), that indicated that the performance of resting mast cells, plasma cells, and M1 macrophages in AAV was antagonistic. The relationship between plasma memory cells and M1 macrophages was synergistic.

### Discovery of core genes

**Fig. 11:**
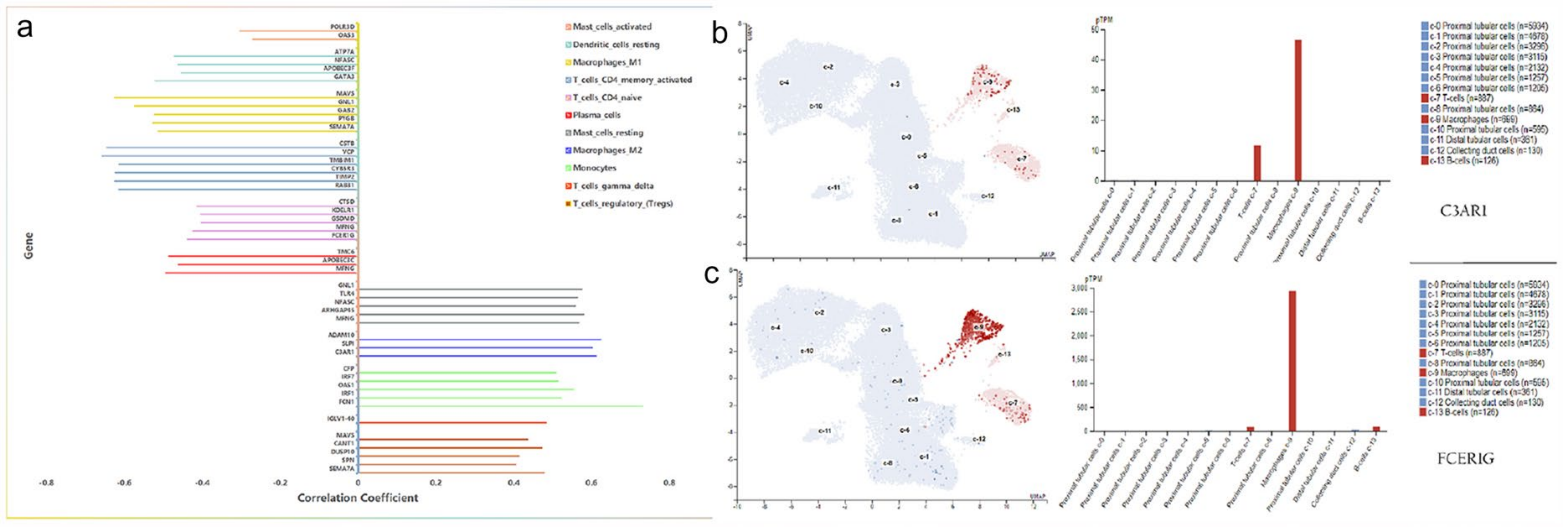
Correlation analysis between key genes in immune effector process and 11 types of immune infiltrating cells and the expression of *C3AR1* and *FCER1G* in the kidney. **a** The vertical items are the names of immune cells. The horizontal items indicate the correlation coefficient (different colors represent different immune cell types). **b** The expression of *C3AR1* in the kidney. **c** The expression of F*CER1G* in the kidney

The correlation between the core genes of the “*immune effector process*” and 11 types of the immune cell infiltration process is shown in Fig. 11a. Among 44 genes that showed close association with immune infiltrating cells, 4 hub genes involved in the “*immune effector process*” in the black module, those associated with differentially immune infiltrating cells were *C3AR1* positively correlated with M2 Macrophages, *FCER1G* negatively correlated with naive CD4 T cells, and no significant correlation was found for the remaining hub genes. Analysis at the human protein atlas website revealed that *C3AR1* and *FCER1G* were significantly highly expressed in C9 Macrophages of renal tissue (Fig. 11b, c).

### Association between hub genes and clinical features of AAV

**Fig. 12:**
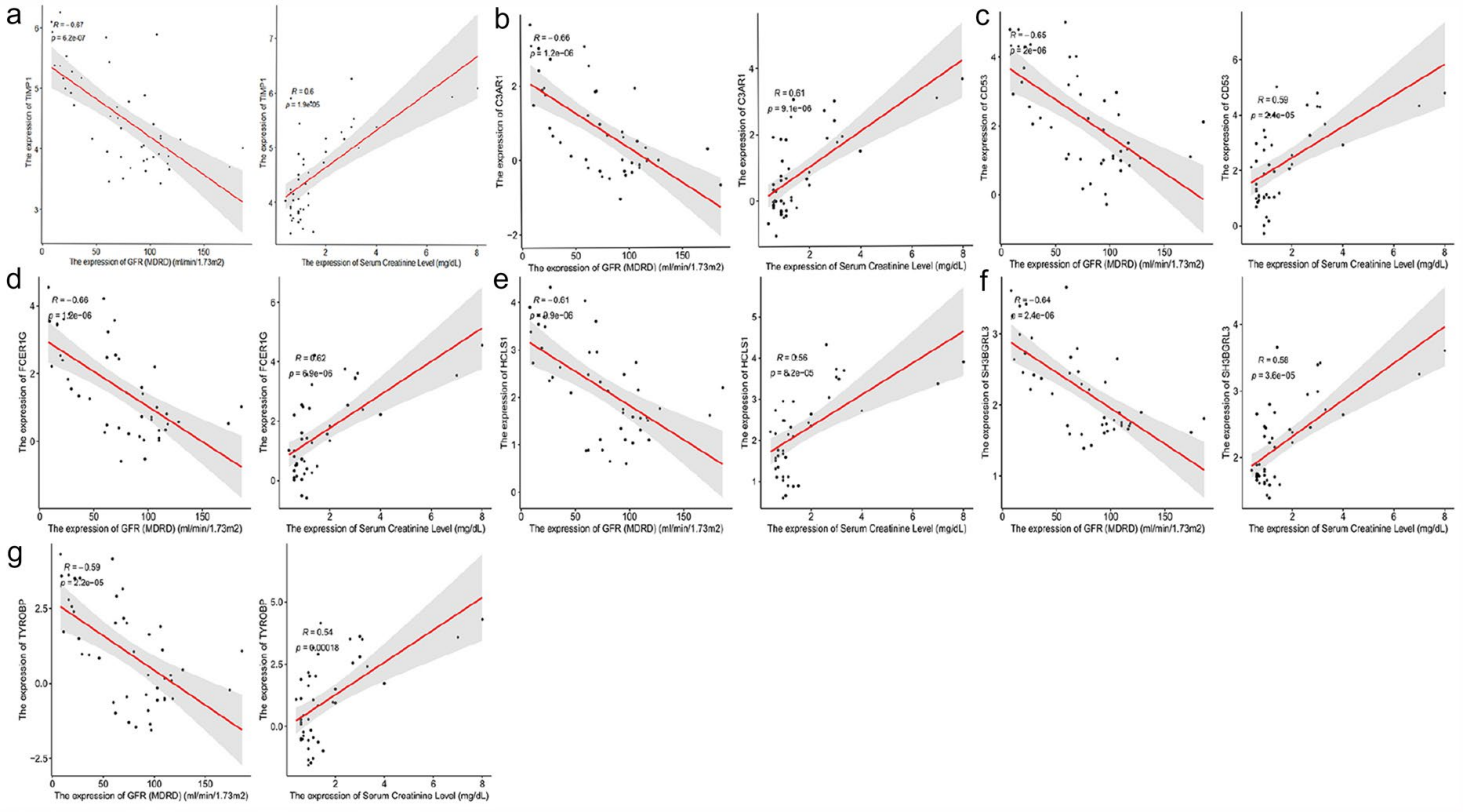
Correlation between mRNA expression of 7 hub genes in glomerulus and GFR, SCr in AAV. *TIMP1* **a**, *C3AR1* **b**, *CD53* **c**, *FCER1G* **d**, *HCLS1* **e**, *SH3BGRL3* **f,** and *TYROBP* **g** correlated with GFR and positively correlated with SCr in AAV

After online “*Nephroseq v5 platform*” analysis, the results showed that mRNA expression of *TIMP1* (Fig. 12a), *C3AR1* (Fig. 12b), *CD53* (Fig. 12c), *FCER1G* (Fig. 12d), *HCLS1* (Fig. 12e), *SH3BGRL3* (Fig. 12f), and *TYROBP* (Fig. 12g) in glomerulus reversely correlated with glomerular filtration rate (GFR) and positively correlated with serum creatinine (SCr) in AAV patients. In this case, upregulated hub genes were required to enhance the implementation of AAV.

### Identification of agents to prevent AAV injury

CMap analytics was used to identify small molecular compounds that reversed the expression of 7 hub genes in cell levels to predict potential medications for the improvement of the disease. The most significant compounds are shown in Table 2.

**Table 2:** Small molecular compounds provided by CMap analysis to reverse expression

Rank	CMap name	Mean	Enrichment	*P*	Percent Non-null
1	Fisetin	− 0.794	− 0.995	0	100
2	5182598	− 0.74	− 0.966	0.002	100
3	Demecolcine	− 0.697	− 0.983	0	100
4	Pnu-0293363	− 0.678	− 0.954	0	100
5	Splitomicin	− 0.636	− 0.967	0	100
6	12,13-EODE	− 0.631	− 0.965	0	100
7	5162773	− 0.548	− 0.929	0	100
8	Cromoglicic acid	− 0.61	− 0.927	0.011	100
9	1,5-isoquinolinediol	− 0.521	− 0.914	0	100

## Discussion

So far, our study is the first to use WGCNA to probe the gene-network signature of kidney tissue to AAV. Two mRNA microarray datasets downloaded from GEO were enclosed for further analyses. DEGs between renal glomerulus tissues of AAV patients and normal controls were screened to work out the hub genes within the most connected module. The study of GSEA examined possible underlying causes of ANCA glomerulus damage. We examined the variance between AAV kidney tissue and normal kidney tissue in immune cell by using CIBERSORT. Furthermore, the Nephroseq v5 online tool was used for correlation study of hub genes and clinical features of AAV. The CMap analysis offered small molecular compounds to reverse altered expression of hub gene in cell lines.

Most patients with AAV have renal involvement, and both patient survival and risk of ESRD are closely related to renal function at the time of presentation [24]. It has long been recognized that both environmental and genetic factors contribute to the development of AAV. Moreover, different types of AAV have genetic differences. Multiple studies found that anti-proteinase 3 ANCA was correlated with *HLA-DP* and serpin A1 gene (*SERPINA1*) and proteinase 3 (*PRTN3*), but anti-myeloperoxidase ANCA was linked with *HLA-DQ*, which depended on antigenic specificity of ANCA [25–27]. Recent clinical and basic science studies have demonstrated the roles of neutrophils, other immune cells, and humoral factors in the pathogenesis of AAV and improved to several breakthroughs in the grasp and treatment of this disease [28]. Although in-depth efforts have been created, the underlying mechanisms of AAV renal injury stay elusive. The utilization of bioinformatics techniques has allowed us to identify important biomarkers in the development and progression of AAV, which may provide new insight into the further study of this disease.

In this study, we detected 445 DEGs between kidney biopsy specimens of AAV patients and normal controls based on two microarray datasets. WGCNA clusters DEGs in the 6 most relevant gene modules. After analyzing the gene significance between each module and AAV, the interaction of the black module is significantly higher than other modules. Enrichment analyses revealed that major genes in this module joined in various immune responses. As antecedently according, immune responses, like the activation of adaptive immune system triggering immune advanced deposition, complement activation, and self-antigen production, displayed a harmful impact on renal glomerular cells, that were related to impaired kidney performance in AAV [29].

A total of 7 DEGs were identified as hub genes, including *TIMP1, FCER1G, SH3BGRL3, HCLS1, C3AR1, TYROBP,* and *CD53*. Among them, *TIMP1* and *FCER1G* were also identified with non-zero regression coefficients as diagnostic markers of AAV by combining LASSO methods. The gene tissue inhibitor matrix Metalloproteinase 1 (*TIMP1*), which encodes a 931 base pair mRNA and a 207 amino acid protein [30], is a tissue inhibitor of Metalloproteinases family. This protein can inhibit the proteolytic activity of matrix metalloproteinases (MMPs) by building non-covalent complexes and control the stability of the matrix reshaping during extracellular matrix degradation [31] and its production increases to antagonize the elevated MMPs expression [32, 33]. It has been confirmed MMPs will be upregulated in inflammation and physiological remodeling processes and implicated in both the inflammatory and fibrotic phase of crescent formation in active renal ANCA-related vasculitis [34], and the increased renal *TIMP1* expression also imaged the inflammatory process in AVV and ANCA-related GN [35]. *FCER1*G is involved in innate immunity [36], which shifted activation signals from various immunoreceptors [37, 38]. *FCER1G* was functionally joined to mediate neutrophil activation and was additionally concerned in platelet activation [39], and improved prognosis by affecting the immune-related pathways in the progression of clear cell renal cell carcinoma (ccRCC). Moreover, *FCER1G* could be a crucial molecule in signal pathways that are wide concerned in an exceedingly sort of immune responses and cell varieties [40]. Combining the results, we speculate that *FCER1G* may play a vital role in the disease progression of AAV given its function in immune regulation. The above analysis shows that AAV can be developed with TIMP1 and FCER1G and that they can also be used as diagnostic markers for AAV. Except the two genes mentioned above, our research demonstrated that the expression level of *SH3BGRL3, HCLS1, C3AR1, TYROBP,* and *CD53* all showed an increasing trend in the AAV kidney tissue compared with normal tissue. By Nephroseq v5 online tool analysis, the upregulation of the above-mentioned 7 hub genes was significantly positively correlated with the GFR and SCr in the progression of AVV. Therefore, we prefer to consider them as an indicator of disease prediction. Although, at present, there is no direct evidence that these 5 genes play a specific role in kidney disease but it provides direction and hypothesis for future AAV gene studies.

To further explore the association between hub genes and immune cell infiltration in AVV, we used CIBERSORT. We found an increased infiltration of resting mast cells, monocytes, regulatory T cells (Tregs), αβ T‐cell, and M2 macrophages, while a decreased infiltration of plasma cells, naive CD4 T cells, activated memory CD4 T cells, M1 macrophages, resting dendritic cells, and activated mast cells may be related to the occurrence and development of AAV. Mast cells are critical antigen-presenting cells that have arisen as potential main players in general and progressively progressive ways of GN activation, amplification, and paradoxically immunomodulation [41, 42]. Confirmed through in vivo experiments that the earlier vasculitis at 48 h was thought to be mast cell dependent [43], and MCs are the largest populations of leukocytes in AAV infiltration of injured kidneys and the most serious tubulointerstitial injury has occurred to patients with higher MC density [44]. The previous experimental studies suggested MCs can improve the development of immunomodulation by interacting with Tregs in AAV [45], which was presented in the form of a strong positive correlation between mast cells and Tregs in our study. In addition, the dominant form of infiltrating cell in the early biopsies of ANCA-associated GN is CD68 + monocytes/macrophages. In this setting, monocytes can be used to orchestrate the immunologic response [46] and triggered in vasculitis [47]. Our results were also consistent with the previously reported skewed balance of CD4 + T cells, including the increase of Tregs and the decrease of naive cells in AAV patients [48–50]. Another interesting finding was that neutrophils had always been thought to be closely related to AVV [51]. However, there were no significant differences in the expression of neutrophils in the present study, which may be related to the formation of neutrophil extracellular traps (NETs), resulting in the number of activated neutrophils had increased in the meantime, while the total number of neutrophils remained almost unchanged [52]. Previous evidence in combination with our findings has shown the importance of resting mast cells, monocytes, regulatory T cells, and activated neutrophils in AAVs and should be the highlight of further research. Furthermore, our results demonstrate 11 types of AAV immune cells. The mast cells are closely linked to the infiltration of regulatory T cells and plasma cells. However, there is no relevant research report on it, further scientific evidence is essential for the basic processes of such associations. In an analysis of the relationship between the Hub genes and immune cells, the *C3AR1* association with the M2 Macrophages was found to be extremely positive. *FCER1G* was significantly negatively correlated with naive CD4 T cells. Studies have shown that M2 macrophages are participated in acute renal injury of glomerulonephritis with crescents especially in AAV [53, 54]. Therefore, combine results about CD4 + T cells indicated that *C3AR1 and FCER1G* may be involved in AAV development by immune regulation. This requires further study in order to clarify the dynamic possible relationship between genes and immune cells.

We uploaded 7 hub genes into the CMap database and matched them with small molecule treatment. As a natural flavonoid, fisetin was confirmed to prevent high fat diet-induced diabetic nephropathy through the suppression of insulin resistance and the inflammation mediated by RIP3 [55], and reduce the inflammation of kidneys and apoptoses by inhibiting the Src-mediated NF-κB p65 and MAPK pathways to guard against the LPS-induced septic AKI mice [56]. As yet, no relevant studies have shown the effect of these compounds on kidney damage in AAV.

Although our study provides new ideas for the diagnosis and treatment of AAV, it still has some limitations. Firstly, as we know, there are three different types of AAV and that they are all genetically different from each other. However, based on the results of our study, no correlation between the above-mentioned genes that distinguish AAV types and immunomodulatory cells was found. For further studies, we will work on an in-depth analysis of the subtle differences between the different types of AAV and verify our current findings and clarify the biological functions of the AAV genes by basic studies. Second, we need to perform in vivo experiments to verify the specific mechanism of action of hub gene with differential immune cell. Third, there are not enough available sequencing datasets for AAV disease model, and we need to further refine the lack of this field.

## Conclusion

Taken together, our study identified *TIMP1* and *FCER1G* had the potential to be used as diagnostic markers for AAV. 11 types of immune cells revealed important associations with AAV, resting mast cells and monocytes showed the greatest variations. In this case, *C3AR1*and *FCER1G* may be involved in AAV development by immune regulation.

## Data Availability

Datasets used and/or analyzed in this study are available from the corresponding author on reasonable request.

## Abbreviations

AAV: Antineutrophil cytoplasmic antibody (ANCA)-associated vasculitis
GEO: Gene expression omnibus
GSEA: Gene set enrichment analysis
LASSO: The least absolute logistic regression of the shrinkage and selection operator
CMap: Connectivity map
DEGs: Differentially expressed genes
GO: Gene ontology
WGCNA: Weighted gene co-expression network analysis
PCA: Principal component analysis
ROC: Receiver operating characteristic
MPA: Microscopic polyangiitis
GPA: Granulomatosis with polyangiitis
EGPA: Eosinophilic granulomatosis with polyangiitis
ANCA-GN: ANCA-associated glomerulonephritis
ESRD: End-stage renal-disease
SAM: Significance analysis of microarrays
FDR: False discovery rate
MM: Module group members
CC: Cellular component
MF: Molecular function
GFR: Glomerular filtration rate
SCr: Serum creatinine
SERPINA1: Serpin A1 gene
PRTN3: Proteinase 3
TIMP1: Metalloproteinase 1
MMPs: Matrix metalloproteinases
ccRCC: Clear cell renal cell carcinoma
NETs: Neutrophil extracellular traps
